# Taking a Tailored Approach to Material Design: A Mechanistic Study of the Selective Localization of Phase-Separated Graphene Microdomains

**DOI:** 10.1021/acsami.4c05666

**Published:** 2024-05-15

**Authors:** Suihua He, Baris Demir, Pascaline Bouzy, Nicholas Stone, Carwyn Ward, Ian Hamerton

**Affiliations:** † Bristol Composites Institute, School of Civil, Aerospace, and Design Engineering, Queen’s Building, University of Bristol, University Walk, Bristol BS8 1TR, U.K.; ‡ Centre for Theoretical and Computational Molecular Science, The Australian Institute for Bioengineering and Nanotechnology, The University of Queensland, Brisbane, Queensland 4072, Australia; § Physics and Astronomy, College of Engineering, Mathematics and Physical Sciences, University of Exeter, Exeter EX4 4QL, U.K.

**Keywords:** reaction-induced phase separation (RIPS), epoxy resin, graphene, microstructure, molecular dynamics simulations

## Abstract

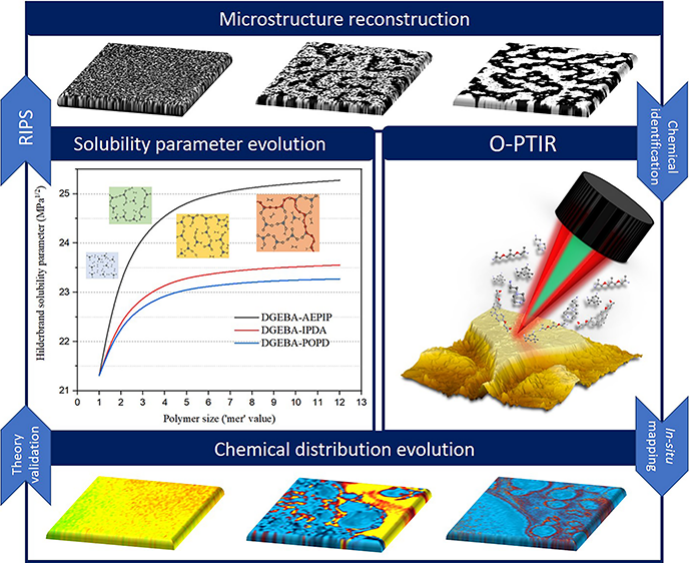

To achieve multifunctional properties using nanocomposites, selectively locating nanofillers in specific areas by tailoring a mixture of two immiscible polymers has been widely investigated. Forming a phase-separated structure from entirely miscible molecules is rarely reported, and the related mechanisms to govern the formation of assemblies from molecules have not been fully resolved. In this work, a novel method and the underlying mechanism to fabricate self-assembling, bicontinuous, biphasic structures with localized domains made up of amine-functionalized graphene nanoplatelets are presented, involving the tailoring of compositions in a liquid processable multicomponent epoxy blend. Kinetics studies were carried out to investigate the differences in reactivity of various epoxy-hardener pairs. Molecular dynamics simulations and *in situ* optical photothermal infrared spectroscopy measurements revealed the trajectories of different components during the early stages of polymerization, supporting the migration (phase behavior) of each component during the curing process. Confirmed by the phase structure and the correlated chemical maps down to the submicrometer level, it is believed that the bicontinuous phase separation is driven by the change of the miscibility between various building blocks forming during polymerization, leading to the formation of nanofiller domains. The proposed morphology evolution mechanism is based on combining solubility parameter calculations with kinetics studies, and preliminary experiments are performed to validate the applicability of the mechanism of selectively locating nanofillers in the phase-separated structure. This provides a simple yet sophisticated engineering model and a roadmap to a mechanism for fabricating phase-separated structures with nanofiller domains in nanocomposite films.

## Introduction

Compared to the phase separation phenomena observed in immiscible polymer blends, which may be initiated from a thermodynamic equilibrium condition, reaction-induced phase separation (RIPS) is more adaptable for thermoset-based polymeric blends.^1−6^ In the past few decades, RIPS has proven to be one of the leading examples of a number of techniques used to improve physical properties, especially fracture toughness of thermosets (TS) by promoting a two-phase morphology.^7,8^ In RIPS, the phase morphology develops during the polymerization of an initially homogeneous mixture containing a TS resin and a second-phase polymeric modifier, such as rubber,^9,10^ thermoplastic,^11−14^ or block copolymer.^15−17^ Reducing the viscosity of a high-performance TS resin by blending with a low-viscosity and easily processable modifier is one of the common methods to increase its processability, and numerous studies have been reported based on investigations of the structure–property relationships in TS/TS blends.^18−20^ However, phase-separated structures arising in TS/TS blends, which might result in some unexpected properties, have rarely been successfully prepared.

The formation of miscible blends, through copolymerization or the formation of interpenetrating polymer networks, and the strong chemical or physical interactions between the different components can be achieved in most TS/TS blends.^21,22^ The initial kinetics asymmetry, as well as the formation of interpenetrating polymer networks, and the copolymerization between two components all hinder phase separation. Hence, it is commonly believed that phase-separated structures can only be induced in a narrow range of compositions,^23−26^ and, unlike thermoplastics (TP),^27^ it is difficult to fabricate a phase-separated structure for TS/TS blends in a controllable manner. For instance, Yue et al.^28^ concluded that, compared with the identification of the phase structures in TP/TP, TP/TS, and rubber/TS blends, analysis of the phase structure in TS/TS blends through chemical means is much more complicated due to the copolymerization between various components.

A few routes to achieve phase-separated structures via RIPS in TS/TS blends have been reported in the past decade, such as benzoxazine/cyanate ester,^26^ benzoxazine/bismaleimide,^4,29−31^ and benzoxazine/epoxy blends.^6,32−35^ Several strategies to achieve RIPS have been investigated to determine the factors influencing phase separation and morphology–structure relationships in TS/TS blends. Wang et al. fabricated benzoxazine/bismaleimide blends with bicontinuous phase structures via imidazole catalysis and found that viscosity was the principal influence on phase separation and phase morphology.^4^ Zhao and co-workers investigated the RIPS behavior in benzoxazine/epoxy systems, and the results showed that sequential polymerization of epoxy and benzoxazine could be effected by increasing the initial molecular weight of epoxy or by adding an imidazole initiator, resulting in a phase-separated structure;^6,32−35^ these materials have been proven to exhibit significantly improved toughness. Achieving selective localization of nanofillers specifically in the phase-separated domain is one of the most promising techniques for fabricating multifunctional materials. Additionally, Huang et al.^36^ reported the successful selective localization of multiwalled carbon nanotubes (MWCNT) in the continuous domain of a TS/TS blend, which resulted in a dramatic improvement in the toughness of the epoxy resin and the electrical properties of the MWCNT/epoxy composites without significant losses in tensile strength and modulus. The interfacial energy between MWCNT and two different types of epoxy resin (DGEBA) and tung oil-based diglycidyl ester was inferred as the mechanism of the selective localization of MWCNT in the TS/TS blend. However, it is believed that during the polymerization of TS blends, the system entropy would decrease before the dynamic equilibrium is re-established, meaning that the corresponding interfacial energy between different components might change accordingly.

Thus, a further exploration and explanation, which is more applicable to TS blends, is worthy of investigation to reveal the underlying mechanism of selective localization of nanofillers in TS blends. We have previously demonstrated a simple and effective route to fabricate a microstructured dielectric nanocomposite film with high thermal conductivity and mechanical properties^37^ and for the first time the possibility of using optical photothermal infrared (O-PTIR) spectroscopy to assist the investigation of the phase separation mechanism of an epoxy blend with selective localization of nanofillers.^38^ Since it was not possible to determine the underlying phase separation mechanism directly through chemical mapping, a series of theoretical studies were performed to reveal the mechanism of phase morphology evolution during polymerization, which might enable the controlling of the phase structure with selective localization of nanofillers in TS systems.

## Experimental Section

### Materials

Component A, RS-M135 (PRF Composites, UK), was an epoxy resin produced from bisphenol A diglycidyl ether (DGEBA) (CAS no. 25068-38-6) with a number-average molecular weight *M*_n_ of <700 g/mol. Component B, RS-MH137 (PRF Composites, UK), was a hardener containing (a) isophorodiamine abbreviated as IPDA (3-aminomethyl-3,5,5-trimethylcyclohexylamine) (CAS no. 2855-13-2, 35–50% w/w) and (b) poly(oxypropylenediamine) abbreviated as POPD (CAS no. 9046-10-0, 50–70% w/w). Component C, 1-(2-aminoethyl)piperazine (AEPIP), was purchased from Sigma-Aldrich (CAS no. 140-31-8). Amine-functionalized graphene nanoplatelets (A-GNPs) with a mean diameter of 2 μm and a thickness under 4 nm were purchased from Cheap Tubes, Inc., USA, and used as reinforcement in this study. All the materials in this study were used as received without further purification. Multiwalled carbon nanotubes (MWCNTs) with an average length of 15–20 μm and a diameter of 8–18 nm were provided by Nanografi (St. Louis, MO, USA).

### Sample Preparation

To process the materials, first, graphene nanoplatelets (3 wt %) were dispersed in the curing agents in the appropriate ratio by a sonication probe in a water bath at room temperature for 1 h. Before the mixing process, the neat epoxy was degassed via a vacuum line at 25 °C for 10 min. Components A and B were mixed to fabricate a composite blend, named A-B. Another set of samples was cured by the hardener with components B and C in 85:15, 8:2, and 7:3 weight ratios, named A-BC (85:15), A-BC (8:2), and A-BC (7:3). The blends were then mixed by using a mechanical stirrer for 10 min at 1000 rpm. Finally, the A-GNP/epoxy mixture was spin-coated on a glass substrate with a rotation speed of 1500 rpm. The composite films were kept at room temperature for 60 min before a postcure process (60 °C for 4 h) was employed. The epoxy resin (A) and hardeners (BC) were mixed in a 10:3 weight ratio for all samples.

### Imaging Characterization

Distribution and dispersion of the GNPs in the epoxy matrix on a larger scale were studied using an optical transmission microscope (Zeiss Axio Imager 2, Carl Zeiss MicroImaging GmbH, Jena, Germany). Representative images were captured and then processed using ImageJ software (https://imagej.net/downloads). Atomic force microscopy (AFM) images were collected using a Dimension XR (Bruker, Santa Barbara) with an Icon scanner operating in the peak force tapping mode (nominal spring constant of 0.4 N/m, peak resonant frequency of 2 kHz). Transmission electron micrographs were obtained on a Tecnai T12 (Thermo Fisher) electron microscope at an accelerating voltage of 120 kV. The transverse sections of samples for electron microscopy were cut via an Ultracut E ultramicrotome. Sections were 80 nm thick and supported on grids coated with a Pioloform film.

### MD Simulations

All-atom MD simulations were performed using the DREIDING force field with the Buckingham potential.^39^ A Nosé–Hoover thermostat^40,41^ and barostat^40,42^ were used to control the temperature and pressure, respectively. A cutoff distance of 12 Å was considered for the calculations of the long-range van der Waals and Coulombic interactions. A tail correction and the particle–particle–particle–mesh algorithm (PPPM)^43^ were used for the calculations of Coulombic interactions between charged atoms. Newton’s equations of motion were time-integrated with a time step of 1 fs. Periodic boundary conditions (PBCs) were applied in all directions. The LAMMPS simulation software package^44^ was utilized to perform MD simulations.

Monomers and hardeners were generated, and geometry was optimized using AVOGADRO software.^45^ The geometry-optimized structures (i.e., a monomer) were placed in a cubic simulation cell with a dimension of 200 Å, and partial atomic charges were calculated following the procedure reported in the literature.^46^ Next, a liquid sample for each system was generated following the experimentally reported compositions, using PACKMOL software.^47^ A polymerization protocol^46^ was applied to generate polymer samples with different degree of conversion (DOC) values. DOC is defined as the ratio between the number of reactive carbon atoms of the monomers and the number of reactive carbon atoms that are initially present in the simulation cell.

### O-PTIR Measurements

O-PTIR measurements were carried out using a mIRage infrared microscope (Photothermal Spectroscopy Corp., Santa Barbara, USA) equipped with a 40× objective (N.A. 0.78) and a four-module-pulsed QCL with a tunable range from 1799 to 785 cm^–1^. The measurement principle of O-PTIR is reported in ref (38). Briefly, in O-PTIR spectroscopy, a tunable quantum cascade laser (QCL) was used to illuminate the sample; when the wavelength of the IR beam matched with a molecular vibrational frequency, the energy generated by the light absorption was converted to heat. A temperature fluctuation resulted in the modulated changes in the volume (photoacoustic effect) and refractive index (photothermal effect), and thus, the IR spectrum of a single spot could be acquired and chemical images with the submicrometer spatial resolution were generated. In this work, single-point spectral data were acquired with a 6.6 cm^–1^ spectral resolution and 9 scans per spectrum. Single IR frequency images were collected in the reflective mode at a 500 nm step size (pixel size) by tuning the QCL device to the frequencies corresponding to the wavelengths of 1512 and 1320 cm^–1^; ratio images were created from the individual scans. Specifically in this work, the investigation of chemical distribution evolutions was done by capturing the chemical map of the specific blend in a different timeslot. Instrument control and data collection were performed using PTIR Studio 4.3 software supplied by the manufacturers.

## Results and Discussion

### Examining Morphology Evolution during Polymerization

**Figure 1:**
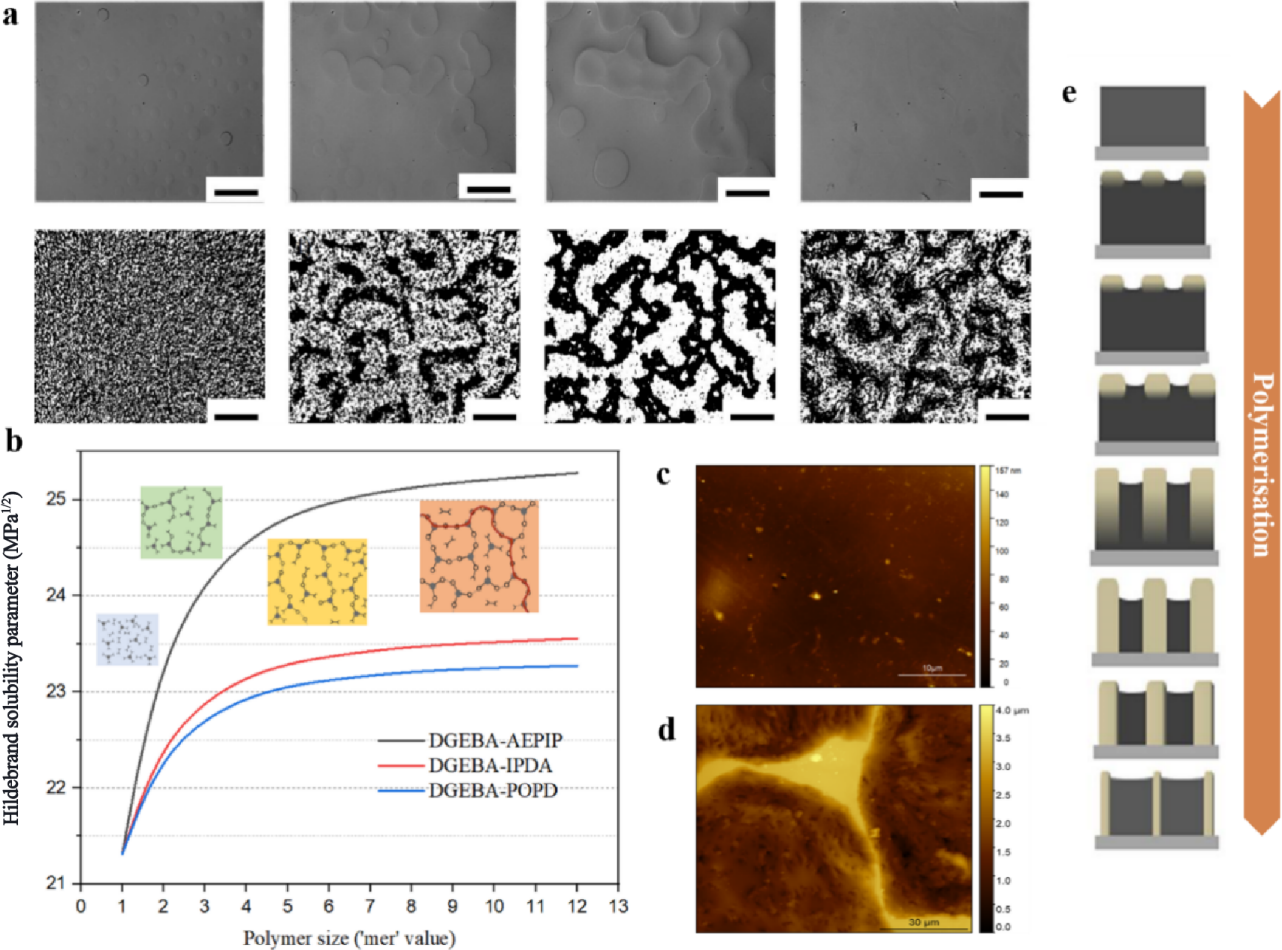
Morphology and solubility parameter development during polymerization. (a) Microstructure reconstruction of the A-BC (85:15) blend without (top) and with (bottom) A-GNPs with a time history of 0, 5, 30, and 180 min (scale bar: 100 μm). (b) Plot of Hildebrand solubility parameters (MPa^1/2^) for different monomers calculated using Fedors’ method as a function of the monomer (polymer size relates to the degree of conversion). (c) AFM image of the epoxy film. (d) AFM image of the nanocomposite film. (e) Schematic representing the vertical cross section during the phase separation process of the nanocomposite blend (bright regions: epoxy domains; dark regions: A-GNP domains).

To achieve a better understanding of the underlying mechanism of phase separation and selective localization of nanofillers in TS blends, the *in situ* microstructure evolution during polymerization was monitored. The combination of this multicomponent blend is a liquid processable commercial formulation, wherein amine-functionalized graphene nanoparticles (A-GNPs) are incorporated as reinforced particles. The A-GNPs are homogeneously dispersed in the epoxy matrix at the initial stage of curing, but the incorporation of 1-(2-aminoethyl)piperazine (AEPIP) into the chemical formulation leads to the self-assembly of a continuous phase-separated domain with graphene nanoplatelets, which was reported in ref (37). Figure 1a highlights the reconstructions of the microstructures observed for A-BC (85:15) during the curing process, showing a sequence of images from initial to cured stages. This unexpected reverse-phase behavior stimulated a more detailed exploration of the phase separation mechanism explained in the following sections. In the initial stage, A-GNPs are observed to be distributed evenly in the epoxy matrix, indicating that the ultrasonic pretreatment effectively disperses them. However, the formation of secondary phase A-GNP domains was observed after the blend was allowed to remain for 5 min at room temperature. Some circular inclusions (A-GNP domains) were formed rapidly from the homogeneously distributed A-GNPs, and then, the rest of the graphene sheets were attracted by the nearby A-GNP aggregates to form bigger aggregates. Furthermore, “necks” were seen to develop between adjacent sunken domains. Surprisingly, a distinct bicontinuous phase-separated morphology appeared at 30 min. Distinct darker and lighter regions could be observed in this stage, which represented the A-GNPs and resin, respectively. Most importantly, after the blends were allowed to remain in an equilibrium state for approximately 10 min, the graphene sheets separated from the interface of “dark” to “bright” domains, leading to the expansion of “dark” domains and narrowing of the “bright” domains. Hence, the highly heterogeneous, spatially distributed, A-GNP domains could be observed owing to the disassociation after the formation window of the phase-separated structure (from 0 to 40 min). Interestingly, the evolution of phase-separated domains in the epoxy blends that lack A-GNPs is consistent with the blend with A-GNPs. This implies that the phase behavior is induced by the reaction of different chemical components during the polymerization (see Figure 1b) but not by the incorporation of A-GNPs. The final topography of the cured films with or without A-GNPs is shown in Figure 1c,d. Figure 1c depicts the comparatively smooth surface (roughness of ∼40 nm) of the homogeneous film. Conversely, in Figure 1d, the nanocomposite film consists of two distinct domains separated by a consistent height step with a surface roughness of ∼2 μm. Accordingly, the morphology evolution of the nanocomposite blend is schematically displayed in Figure 1e, representing the corresponding phase behavior during the phase separation.

### Mechanistic Investigations of the Phase Behavior via RIPS

It has been reported that the distribution of nanofillers in immiscible polymer blends is affected by the interactions between the different components. Thus, the wetting coefficient has been used to predict the distribution of nanofillers in immiscible polymer blends,^11−14^ or even initially miscible TP/TS systems.^48^ To calculate the wetting coefficient, a binary polymer blend is necessary to identify the relative differences in the affinity between the nanofillers and the different types of polymers. However, in this current work, phase separation occurs in the blends containing only DGEBA as the matrix, while the hardener is varied. Thus, it is obvious that the wetting coefficient is not a reliable predictor of the selective localization of TS blends containing a single matrix.

As a result, to determine the influence of the dissimilarity in miscibility between different components on the phase behavior, the solubility parameter of each component is to be calculated. The Fedors method^49^ was used, as this is a simple and straightforward calculation that could be taken as an initial guide to the ambient temperature compatibility of the components of the blend and combined with δ = (Δ*E*_v_/*V*)^(1/2)^ as a simple single temperature (25 °C):
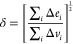
1where Δ*e*_*i*_ and Δ*v*_*i*_ are the additive atomic group contributions for the energy of vaporization and the molar volume, respectively, at a given temperature. As an example, fractional contributions of AEPIP are summarized in Table S1. This additive method produces a calculated solubility parameter, δ, which depends on the nature of functional groups within the monomer/polymer blend structure, as summarized in Table S2.

To determine the evolution of solubility parameters during polymerization of amine-cured epoxy resin blends, Mezzenga et al.^50^ reported a straightforward approach that relied on Kiefer’s assumption,^51^ which was a linear relationship for the various contributions to the solubility parameters in conversion. On this basis, Figure 1b shows the effect of network growth on the solubility parameter calculated using Fedors’ method for the multicomponent blends. This indicates that in a reactive TS system, the miscibility between each component is bound to change as the reaction proceeds. It should be noted that the solubility parameter calculated for the fragments constructed with AEPIP increases rapidly during the early stage of polymerization and then reaches an equilibrium state after the formation of the pentamers. In comparison, the pairs constructed with isophorodiamine (IPDA) and poly(oxypropylenediamine) (POPD) presented similar solubility parameters over time, and both remained much lower than the pairs containing AEPIP. Therefore, RIPS, rather than the initial miscibility between components (Table S2), is believed to be the driving force for the phase separation in multicomponent epoxy blends and most importantly for the selective localization of A-GNPs in the nanocomposite blends.

### Curing Kinetics

Gu and co-workers^4,26,30^ found that the phase morphology of RIPS would vary due to the dissimilarity in curing kinetics and the corresponding differences in the mobility of various reaction pairs. This was ascribed to the structural differences among various components in the polymer matrix, which played a key role in determining the phase morphology. To identify the nonsynchronous reactions in this multicomponent blend, the reaction subsequence and reaction rate of various reaction pairs were accessed via isothermal and nonisothermal reaction kinetics studies. As shown in Table S3, the calculated rate constants of each combination highlight the reaction speed between different components in various reaction processes, indicating the reaction probability between each component. It is evident that the reaction subsequence of different reaction pairs would probably be DGEBA-AEPIP > DGEBA-IPDA > DGEBA-POPD. It is worth noting that the reactions between DGEBA/AEPIP and DGEBA/IPDA predominantly occur at ambient temperature. As the epoxy-amine reaction is a multistep reaction, peaks were fitted to the DSC exothermic plots, and details of the multistep reaction could be seen from the results of peak deconvolution (Figure S2). The initial stages of each reaction are drawn out, so that several consecutive reactions occur, where the first (and smaller second) peak is associated with ring-opening; the second peak, which accounts for the bulk of the reaction, is associated with bridge-forming.^52^ It is apparent that AEPIP represents an outstanding initiator for the ring-opening process, especially when the thermal initiation is not significant. However, it can be seen that IPDA does not have a significant effect on instigating the ring-opening process, with bridge-forming contributing the main enthalpy for the curing reaction. In contrast, for the blend with POPD, the ring-opening process is much lower, with the etherification reaction being the main reaction responsible for polymerization. In addition, the isothermal curing behavior was analyzed to validate the cure kinetics model, which could most accurately capture the resin curing behaviors during polymerization (Figure S3). It is evident that the reaction rate first increases and then decreases after reaching a maximum rate, which is attributed to the fast ring-opening process caused by AEPIP, and thus, the rapidly increased viscosity hindered the mobility of the molecules; this observation agrees with the nonisothermal measurement. Meanwhile, the following reduction is due to the dramatically increasing viscosity caused by the reaction, which then hinders the mobility of molecules for the reaction.^53^

Regarding the blend containing AEPIP, the reaction rate was much higher than the other blends (all are formed with the amine in excess). For this autocatalytic reaction, the reaction rate first increased and then decreased after passing through a maximum, which is attributed to the fast ring-opening process caused by AEPIP, and thus, the rapidly increased viscosity hindered the mobility of the molecules.^53^ This observation agrees with the nonisothermal measurement in the SI. For the blends containing IPDA and those containing POPD, the reaction rates were very low (three times lower than blends containing AEPIP) and remained stable during the polymerization process. It is hard to initiate the ring-opening process in the presence of the less reactive hardeners at ambient temperature, and thus, the bridge-forming process was delayed. Considering the blends containing AEPIP/IPDA/POPD, the reaction rate decreased with the process of polymerization, presumably due to the rapid consumption of AEPIP and the participation of IPDA in the subsequent bridge-forming process.

### MD Simulation

**Figure 2:**
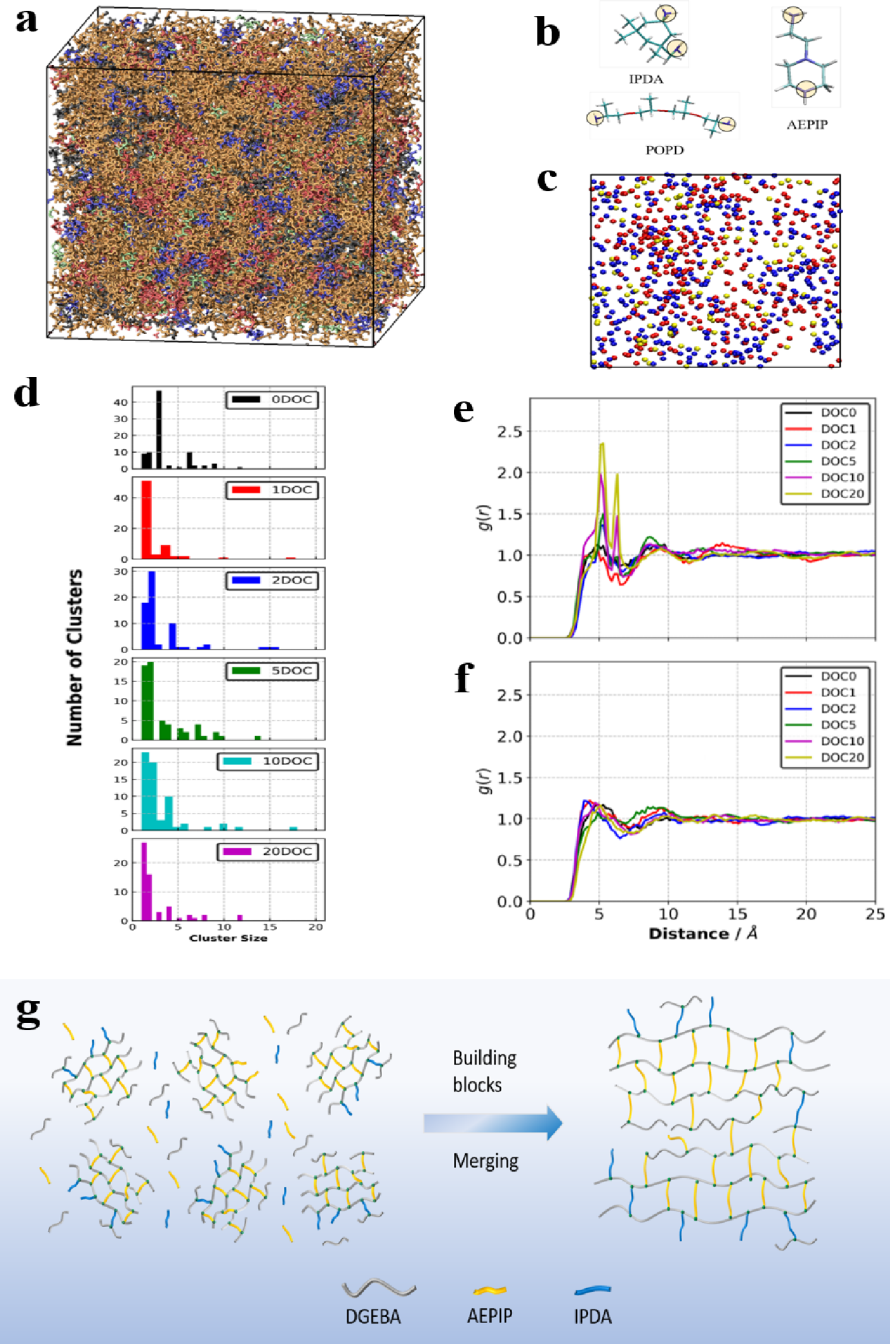
MD simulation results. (a) Schematic of the MD simulation system. (b) Reactive nitrogen sites (blue atoms that are encircled by yellow circles) of various hardeners. (c) Reactive nitrogen sites in the blending system with α = 0.20 (each point represents a reactive site of a hardener; yellow: AEPIP, blue: IPDA, red: POPD). (d) Frequency versus cluster size for AEPIP in the blend as a function of α where the cutoff is 10 Å (DOC represents the degree of conversion). (e) Radial distribution functions (RDF) for the reactive nitrogen sites between IPDA and AEPIP. (f) RDF between the reactive nitrogen sites between POPD and AEPIP. (g) Schematic of migration behavior of molecules during the early stages of polymerization.

To better understand the migration behaviors of molecules in the multicomponent blends, all-atom MD simulations were used to model the blends based on cure kinetics data. Figure 2a shows the 3D resolution of the molecules in the initial liquid state, and the chemical structures of the three different hardeners are shown in Figure 2b. Cluster analysis, which illustrates the number of clusters formed by the aggregation of reactive nitrogen sites of hardeners (Figure 2c), was performed for blends containing AEPIP. As shown in Figure 2d, the various cluster sizes of AEPIP at the initial stage indicate that the AEPIP is randomly dispersed in the blend. As polymerization progresses, the model suggests that AEPIP appears to undergo aggregation from a big cluster at an early stage of the reaction, especially from degrees of conversion (α) 0 to 0.1. This implies that there is a rapid reaction between AEPIP and DGEBA and thus the construction of DGEBA-AEPIP building blocks. Radial distribution functions (RDF) can provide the probability of finding an atom type A around an atom type B (Figure 2b,c), which is an effective way to track the migration of each component during the curing process. Values larger than 1 (at lower distances) mean that there is a larger probability of finding atom type A around atom type B, compared to that found in the bulk (at larger distances). From the RDF results (Figure 2e,f), the peak amplitudes for the AEPIP-IPDA pair increase rapidly during the polymerization, indicating that the IPDA starts to get closer to AEPIP when α = 0.02 to 0.05. The presence of increasing amounts of IPDA around the AEPIP could be detected during the polymerization, indicating that IPDA would participate in the partially cured region that has been induced by AEPIP. In contrast, the peak amplitudes for the AEPIP-POPD pair remain unchanged during the early stages of polymerization, suggesting that the formation of DGEBA-AEPIP does not force the migration of POPD. These results agree with the aforementioned kinetics studies, which indicated that IPDA would participate in the network formation once the ring-opening process was induced by the AEPIP, while POPD would not participate in the reaction during the early stages of polymerization.

Combining the solubility parameter calculations, kinetics studies, and MD simulations, Figure 2g depicts the migration trajectory of each component during the early stage of network formation, which then drives the formation of phase-separated microdomains. From the initial state of polymerization, some low-molecular-weight DGEBA-AEPIP fragments rapidly form. Owing to the ring-opening process induced by AEPIP, the IPDA would then surround the partially cured region and participate in the reaction and network-forming process. At the same time, the dissimilarity in the miscibility between partially cured domains and uncured domains increases. As a result, the partially cured (DGEBA-AEPIP) domains would aggregate to form a larger domain, and thus, the phase-separated domain is constructed in the microscale. This was confirmed by the empirical observations of the phase behavior of the multicomponent blend during the early stages of polymerization (Figure 1) where some smaller phase-separated domains were first constructed and then gradually aggregated to from the microdomains.

### O-PTIR Measurement

Prior to identifying the chemical dissimilarities of each domain by O-PTIR spectroscopy, bulk IR spectra were obtained (Figure S4). Based on those IR spectra, the characteristic 1512 cm^–1^ band of DGEBA was used as the spectral signature of the epoxy resin in these blends, and the vibration of the secondary or tertiary amine within AEPIP (1280–1320 cm^–1^) was used to confirm its presence, as distinct from the other two amine reagents of component B.

**Figure 3:**
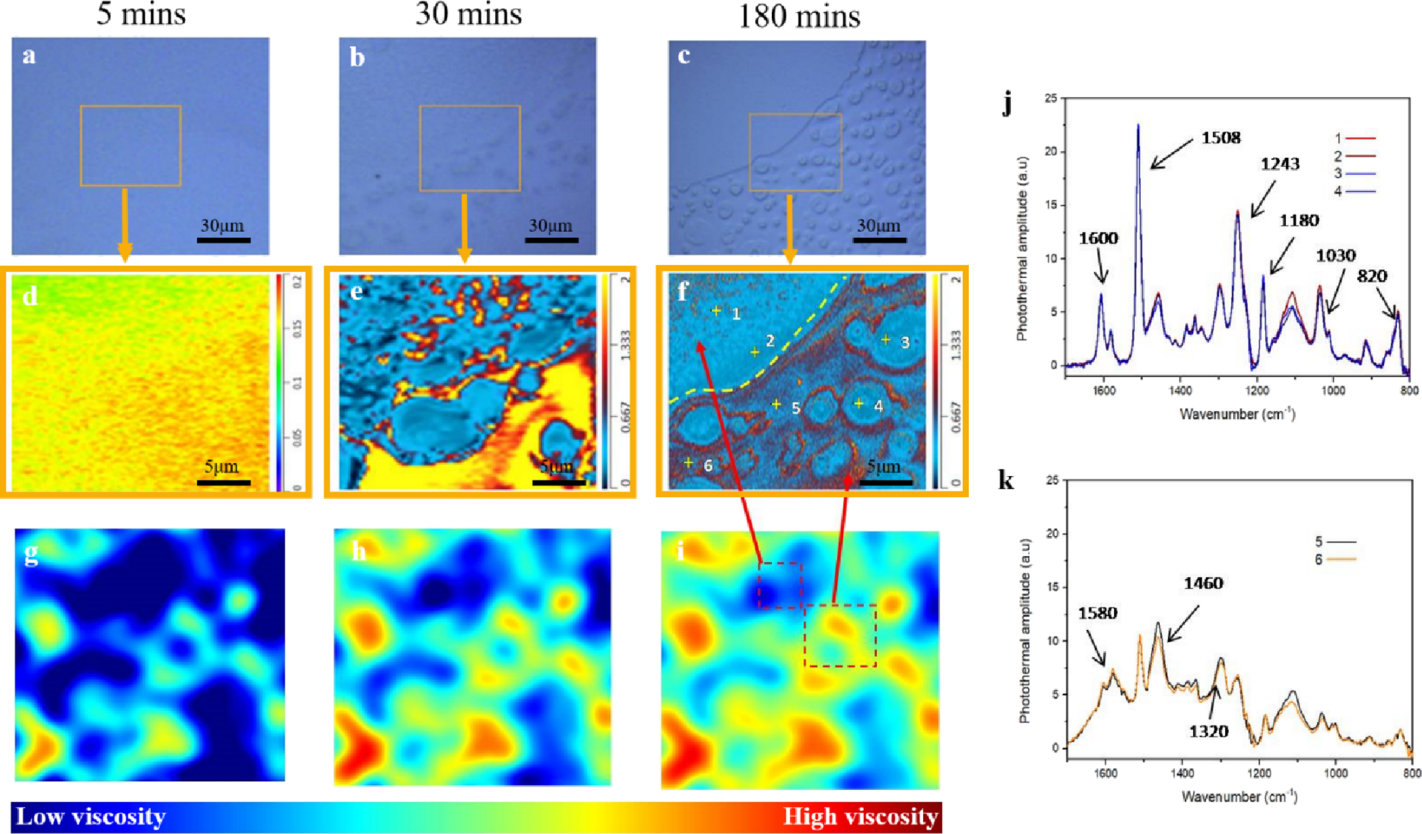
Development of the microstructure and component distribution and the related IR spectra of the A-BC (7:3) blend without A-GNPs. (a–c) Optical images, (d–f) chemical maps (1320/1512 cm^–1^) generated from single frequency O-PTIR images (with 500 × 500 nm^2^ pixels), and (g–i) schematics of viscosity during polymerization (time history: (a) 5, (b) 30 min, and (c) 180 min). (j,k) O-PTIR spectra of different phase-separated domains.

As shown in Figure 3a, it was found that there is no phase separation in the matrix just after spin coating, and the correlated chemical map (Figure 3d) displays a homogeneous compositional distribution with subtle differences in IR absorption in various regions. In Figure 3b,e, it is apparent that a phase-separated structure emerges as the polymerization proceeds, with the chemical map showing significant differences in different regions. This implies that the phase-separated domains were induced by the formation of different fragments in the matrix, which is consistent with the aforementioned results and investigations of the MD section. Most importantly, as shown in Figure 3c,f, the phase-separated structures become more obvious. Conversely, the dissimilarity in the IR absorptions between the different domains dramatically decreases. This is due to the regions displaying an absorption of 1320 cm^–1^, which represents that the secondary amine is increasing. Figure 3j shows the IR spectra of different phases in the final structure; a second phase separation (ca. 2 μm) could be detected by observing the chemical mapping, and its related chemical structure is similar to the epoxy-rich domain. Figure 3k shows that there are a relatively lower IR absorption of 1512 cm^–1^ (aromatic ring) and higher IR absorptions around 1580 (N–H deformation of secondary amine) and 1460 cm^–1^ (C–N stretching) in the domains displaying a higher absorption ratio of 1320/1512 cm^–1^. Comparing the spectra of two different phases confirms that partially cured regions (higher secondary amine) formed by rapid reactions between AEPIP and DGEBA would drive the remaining unreacted epoxide groups from the AEPIP domains due to the discrepancies in miscibility, and thus, the epoxy resin migrates to form the upper layer.

Hence, it can be concluded that the rapid aggregation of AEPIP or the reaction between AEPIP and DGEBA had driven the phase separation; alongside the participation of relatively low reactive hardeners (IPDA and POPD) in the reaction, this resulted in the generation of secondary amine and thus the reduction of chemical dissimilarity of various domains in the final structure (Figure 3d–f). These *in situ* chemical maps provide direct evidence of the relationship between the migration behavior of the components and the microstructure reconstruction, offering the potential to yield an approximation or general prediction of the morphology from the combination of the empirical results presented. To obtain a robust and accurate design of the phase-separated structure, the development in local viscosity of various domains should also be considered, as it has significant effects on the phase behavior of each fragment. However, and to the best of the authors’ knowledge, there are no existing techniques presently available that could detect the *in situ* viscosity with microlevel resolution. Hence, the viscosity of various amine-cured blends during the early stage of polymerization was instead accessed (Figure S5). As shown schematically in Figure 3g–i, the gradually increasing viscosity of the phase-separated domains and the decreased dissimilarity in miscibility between different phases drive the aggregated A-GNP to migrate from the center of the dark domain to the edge of the bright domain, leading to the dissociation of the phase structure of the dark domain.

Based on the aforementioned kinetics studies and MD simulations, the data suggest that the coreaction between the DGEBA-AEPIP would first occur within the reaction mixture. Combining the solubility parameter calculation and *in situ* chemical maps, it is expected that the nanofiller would be prone to locate initially around the DGEBA-AEPIP fragment. Naturally, the viscosity would increase due to the increase in the polymer size. Thus, the heterogeneous reaction between different reaction pairs and the highest reactivity of the DGEBA-AEPIP among the reaction pairs would rapidly increase the viscosity of the specific domain resulting from this coreaction and thus decrease the mobility of the components around the initially polymerized fragments by AEPIP. Consequently, the A-GNPs that are first located in the miscible domains (DGEBA-AEPIP fragments) would be sterically isolated due to the rapidly increasing local viscosity of those domains. This redissociation phase behavior is confirmed in the following section.

### Selective Localization of Nanofillers

**Figure 4:**
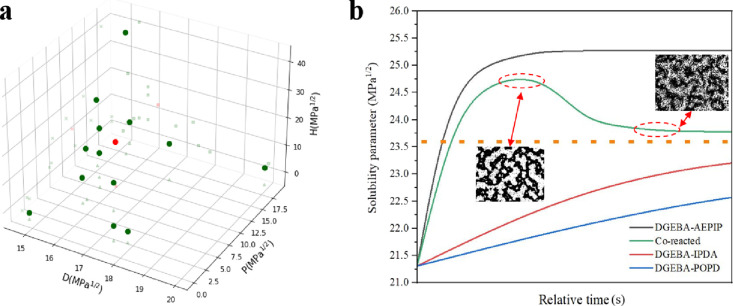
Calculation of phase behavior. (a) Plot of the solubility parameter of A-GNPs (red dot) and solvents (green dots). (b) Plot of solubility parameters (MPa^1/2^) of various fragments as a function of time (the orange dashed line represents the solubility parameter of A-GNPs).

In order to examine the selective localization of nanofillers in the phase structure, their solubility parameters were expected to provide a useful framework to understand and predict the compatibility between different polymeric phases and A-GNPs. Due to the lack of data for the solubility parameters of A-GNPs, the Hansen solubility parameter of A-GNP was determined using UV–vis absorbance measurements,^100^ detailed in the accompanying SI. Hansen solubility parameters for the organic solvents and A-GNPs are shown in Figure 4a,^101^ and the correlated distances between the solubility parameters of the A-GNP and solvents are summarized in Table S2, where the solubility parameter of A-GNPs was calculated as 23.6 MPa^1/2^. Similarly, Hernandez et al.^54^ measured the dispersibility of pristine graphene in 40 solvents and concluded that good solvents for pristine graphene were characterized by having a Hansen solubility parameter close to 23 MPa^1/2^.

To derive an intuitive relationship between A-GNPs and various fragments during polymerization, a further calculation with the consideration of the relation between the degree of conversion versus time and reaction rate of each pair (Figure S4) was performed to form the plot of the solubility parameter versus time (Figure 4b). Based on isothermal DSC measurements made within the window in which phase structure formation takes place (from 0 to 60 min) (Figure S3b), the average reaction rates were obtained as 0.0005 s^–1^ for DGEBA-POPD, 0.001 s^–1^ for DGEBA-IPDA, 0.0025 s^–1^ for DGEBA-Multi, and 0.0045 s^–1^ for DGEBA-AEPIP. Thus, the reaction rate of DGEBA-POPD was denoted as 1R, DGEBA-IPDA as 2R, DGEBA-Multi as 5R, and DGEBA-AEPIP as 9R. The polymer size was then divided by the reaction rate to convert into the time domain. Similar results were obtained from Gu et al.^4,26,30^ They concluded that the sequential reaction would lead to the molecular weight of one component increasing to incompatible in thermodynamics, resulting in the formation of a phase-separated structure. However, in this current work, this method could not explain the “construction and dissociation” phase behavior (Figure 1a). Therefore, the nonsynchronous reactions seem to be sufficient but not necessary conditions for constructing a phase-separated structure in TS blends.

When the solubility parameter of A-GNPs (23.6 MPa^1/2^, orange dashed line) is compared to various fragments during polymerization in Figure 4b, it is found that the A-GNPs should be more compatible with fragments constructed with AEPIP in the early stages of polymerization due to the rapidly increasing solubility parameter of those fragments. As polymerization proceeds, the fragments with AEPIP at their center, either AEPIP-dominated or copolymerized blends (Figure 2g), become incompatible with A-GNPs. Conversely, the DGEBA-IPDA fragment becomes more compatible with A-GNPs due to the gradually increasing solubility parameter during polymerization, thus creating the observed reverse migration behavior. Thus, the A-GNPs would be finally located at the interface between AEPIP-dominated and DGEBA-IPDA regions. Therefore, it could be concluded that the localization of the nanofiller mainly depends on the miscibility discrepancy between different fragments in the TS blends.

**Figure 5:**
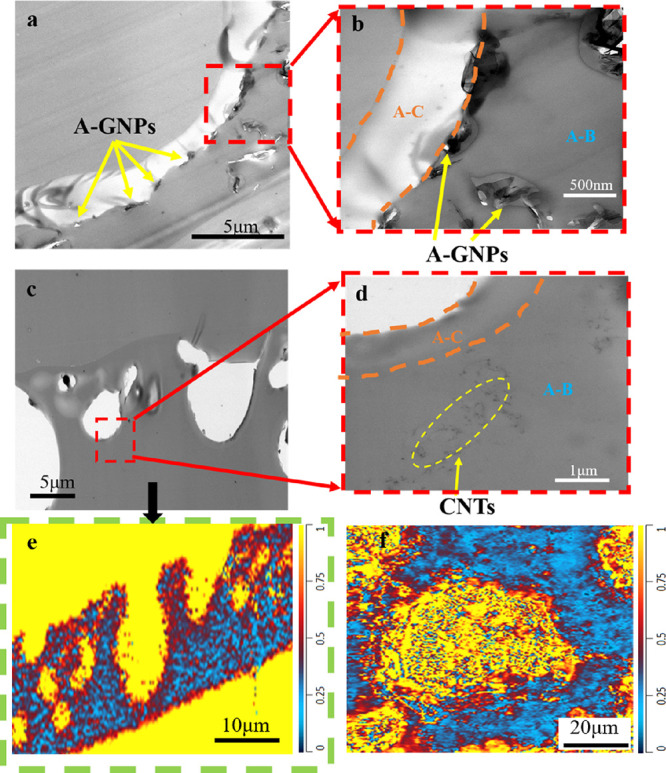
Validation of the selective localization of nanofillers at the submicrometer level. TEM images of (a,b) epoxy/A-GNP blend and (c,d) epoxy/CNTs blend with a BC ratio of 7:3. Chemical map (1320/1512 cm^–1^) of the nanocomposite film, (e) cross section, and (f) top surface. (A-C = DGEBA-AEPIP; A-B = DGEBA-IPDA/POPD).

Multiwalled carbon nanotubes (MWCNT) with a solubility parameter of 22.6 MPa^1/2^^55^ were selected for experimental validation. Based on the calculated solubility parameter evolution of different fragments, the MWCNT were predicted to have a different phase behavior compared to A-GNPs (23.6 MPa^1/2^) in this multicomponent blend. A-GNPs would be located in or around the DGEBA-AEPIP domains, while MWCNT would be prone to locate more remotely from the DGEBA-AEPIP domains. These results were confirmed by the nanofiller localization shown in Figure S6. In order to further evaluate the localization of these nanofillers at the submicrometer level, TEM characterization was performed. Associating TEM images with the chemical maps, it could be confirmed that the A-GNPs were mainly located at the interface between the DGEBA-AEPIP (orange region in Figure 5a,b) and the rest of the domains (blue region). Comparatively, as shown in Figure 5c,d, it is evident that the CNTs were located remotely from the DGEBA-AEPIP domain, as no CNTs could be found around the interfaces. In Figure 5e,f, the chemical maps show that the small domains (voids) are surrounded by the components with stronger absorption at 1320 cm^–1^, which implies that the phase structure is mainly induced by the reaction between different components, alongside the migration of the nanofillers. To be specific, owing to the similarity in the solubility parameter within the initial state of polymerization, A-GNPs preferred to locate in the domains of AEPIP (with more reactive amine groups) cross-linking with DGEBA, which served to sterically trap A-GNPs due to increasing viscosity in these areas during polymerization. Similarly, the mechanics for locating MWCNTs, which display a solubility parameter that is similar to DGEBA-IPDA domains and incompatible with the DGEBA-AEPIP domain, was confirmed by the TEM images with the O-PTIR maps. Hence, it could be concluded that the bicontinuous phase separation is driven by the change of the miscibility between various building blocks forming during polymerization, resulting in the formation of phase-separated microdomains with selective localization of nanofillers.

## Conclusions

In this study, we have demonstrated that nanoparticles can be successfully located in self-assembled microstructures from an entirely miscible blend in a controllable manner. By combining solubility parameter calculations with kinetics studies, the phase separation mechanism of compositions during polymerization has been revealed, which provides a feasible route to realizing the preparation of TS/TS blends with different selective localized nanofillers. This in turn unlocks a promising technique for fabricating nanocomposites with multifunctional properties.

Researching the phase separation phenomenon in TS/TS blends from monomers to oligomers, the dissimilarity in miscibility of the various polymerized fragments induced by heterogeneous reactions was found to be the crucial factor for the phase separation and the selective localization of nanofillers. This was confirmed by kinetics studies, MD simulation, and *in situ* O-PTIR measurements. Specifically, MD simulation revealed the trajectory of different components, supporting the theoretical prediction of the development of solubility parameters and morphology during polymerization. The composition of each domain was identified owing to the high-resolution O-PTIR mapping, providing critical indications that the change of dissimilarity of chemical structures between the developing building blocks would determine the final morphology. The association of TEM images and chemical maps further validated the mechanism of this unexpected phase behavior for designing the phase structure of the thermoset blends with the localization of various nanofillers.

Some limitations on the knowledge of the local viscosity of different domains during the polymerization remain, and so, further exploration is required to establish a more accurate quantitative method from the presently qualitative predictions. Establishing a fully resolved phase diagram is also required, as the closed-loop investigations within have only demonstrated (for the first time) the underlying mechanism for controlling the phase separation of the TS blend with selective localization of nanofillers in a single system. Completion of this further work will provide the probability to design the materials with desired properties by locating nanofillers into the specific domain, thus unlocking the potential to fabricate the next-generation materials in a controllable manner.

## Supporting Information Available

Equations for reaction kinetics studies, evaluation of Hansen solubility parameters, FTIR spectra for all compounds, and additional characterizations and experimental details and methods (PDF)
