# Supplementary material for: Taking a Tailored Approach to Material Design: A Mechanistic Study of the Selective Localization of Phase-Separated Graphene Microdomains
Source: ACS Appl Mater Interfaces. 2024 May 15;16(21):27694–704. doi: 10.1021/acsami.4c05666 (PMC11145585; doi:10.1021/acsami.4c05666)
Supplement: Supplementary file 1 — am4c05666_si_001.pdf [file am4c05666_si_001.pdf]

Taking a tailored approach to material design: a mechanistic study of the selective localization of phase-separated graphene microdomains

Suihua He[1,a], Baris Demir[2], Pascaline Bouzy[3], Nicholas Stone[3], Carwyn Ward[1,b], Ian Hamerton[1,]*

[1]Bristol Composites Institute, Department of Aerospace Engineering, School of Civil, Aerospace, and Design Engineering, Queen's Building, University of Bristol, University Walk, Bristol BS8 1TR, UK

[2]Centre for Theoretical and Computational Molecular Science, The Australian Institute for Bioengineering and Nanotechnology, The University of Queensland, Brisbane, Queensland 4072, Australia

[3]Physics and Astronomy, College of Engineering, Mathematics and Physical Sciences, University of Exeter, Exeter EX4 4QL, UK

[a] present address: Advanced Material Trust, Function Hub, Hong Kong University of Science and Technology (Guangzhou), Guangzhou, 511400, China

[b] present address: School of Engineering, Modelling and Simulation Group (EMSG), UWE Bristol, Frenchay Campus, Coldharbour Lane, Bristol, BS16 1QY, UK

* Corresponding author (ian.hamerton@bristol.ac.uk)

Differential Scanning Calorimetry (DSC)

In this study, DSC experiments were conducted using a Netzsch DSC 204F1 with aluminium pans as sample holders, and sample masses of ~5 mg (uncured samples) and 5.1 ± 0.5 mg (cured samples). Samples were equilibrated at 25°C for 3 mins before cooled to -50°C immediately and held isothermally for 5 min, then heated to 250 °C at a heating rate of 10°C min^{-1} under a constant nitrogen flow of 50 cm^3 min^{-1}. Heat/cool/heat scans, with a cooling rate of 20°C min^{-1}, were used to evaluate the residual curing exotherms. Heat scans were performed with heating rates of 1, 2, 5, and 10°C min^{-1}. In addition, to determine the cure degree over time, isothermal tests were performed at 25°C.

Non-isothermal reaction kinetics

Figure S1 shows the DSC thermograms of various reaction pairs of the multi-component amine-cured epoxy resin blends from the dynamic DSC experiments conducted at heating rates of 1, 2, 5 and 10°C /min, respectively. As expected, the exothermic peak shifts to a higher temperature and the peak width is extended with an increased heating rate. Additionally, the total heat release of the cure reaction increases gradually with an increasing heating rate [1]. Cure kinetics were evaluated using the equation that is better suited to an autocatalytic system than an nth-order model [2]. Therefore, first-order reaction kinetics are assumed, and the Kissinger method [3] used as a preliminary assessment of the resin kinetics for the three main reaction peaks of each blend. Kissinger method (Equation 1) is used, the activation energy and the pre-exponential factor were calculated as below [4],

$$ln\left(\frac{\beta}{T_{max}^2}\right) = ln\left(\frac{AR}{E_a}\right) - \frac{E_a}{RT} \quad (1)$$

where β is the heating rate, T_{max} is the exothermic peak maximum (K); E_a is the activation energy, which is the energy barrier which the reaction must overcome for the reaction to proceed; A is the pre-exponential factor, which is interpreted as the frequency of collisions between reactant molecules at a standard concentration (leading to a reaction or not) per second; and R is the gas constant (8.314 J K^{-1} mol^{-1}).

By plotting lnβ = $(T_{max})^2$ *versus* $1/T_{max}$, the activation energy and pre-exponential factor can be determined from the gradient and y-intercept respectively [4]. The Kissinger method also enables the rate constant (k) for the reaction, which is interpreted as the frequency of collisions between molecules that result in a reaction, to be determined, using the Arrhenius equation expressed as [5]:

$$k = Ae^{\left(\frac{-E_a}{RT}\right)} \tag{2}$$

where, k is the rate constant (s^{-1}) and T is the temperature of the reaction (here 298 K or 25°C was selected as this was the cure temperature used throughout the study). The calculated values are given in Table S4.

Isothermal reaction kinetics

In dynamic DSC experiments, the Kissinger method is not able to evaluate the degree of cure as the reaction progresses, but rather enables certain cure kinetic parameters to be accessed by assuming first-order cure kinetics [6]. Thus, to establish the relationship between phase behaviours and cure behaviours, the isothermal cure kinetics have to be evaluated to investigate the cure behaviour (kinetics) of the resin. Consequently, isothermal DSC measurements were performed to determine the reaction rate and degree of conversion. Assuming that the cure rate of the blending system, $d\alpha/dt$, is proportional to the rate of heat flow, dH/dt, the cure rate can be expressed as [7]:

$$\frac{d\alpha}{dt} = \frac{1}{H}\frac{dH}{dt} \tag{3}$$

The reaction rate *versus* time for the isothermal cure at the ambient temperature of various combinations is shown in Figure S2.

In addition, the degree of conversion of the resin at a given time could be obtained by integrating the area under the curve of cure rate versus time, which is given as [7]:

$$\alpha = \frac{1}{H_U}\int_o^t \left(\frac{dH}{dt}\right) dt \tag{4}$$

The degree of cure versus time for the isothermal cure at the ambient temperature of various combinations is shown in Figure S2.

Fourier Transform Infrared Spectroscopy (FTIR)

PerkinElmer Spectrum 100 FTIR spectrometer (Beaconsfield, UK) was employed to acquire the bulk infrared spectra of the A-GNPs and each component of the resin formulation. The spectrum range was 4000-600 cm^{-1} and 16 scans were acquired and co-added for each measurement. Before conducting measurements, a background scan was run to remove any interferences caused by ambient atmosphere.

Rheological Analysis

Rheological analysis was conducted using a TA Discovery HR-1 hybrid rheometer with a parallel plate fixture, using pairs of disposable aluminium plates (diameter 25 mm, gap 0.3 mm). To determine how the viscosity of various blends changed over time, isothermal tests were performed at 25°C with a shear rate of 5 s^{-1}. The strain frequency used was 1 Hz, and an oscillation amplitude of 50% was selected to match with linear viscoelastic regime [8].

UV-Visible absorbance

Dispersibility of the amine-functionalized graphene nanoplatelets (A-GNPs) in solvents was confirmed *via* UV-Vis absorbance spectroscopy using the Bristol-Xi'an Jiaotong approach reported by the Faul group [9]. A-GNPs solutions were prepared by placing A-GNPs (5 mg) in different solvents (20 mL), using a sonication probe for over 5 min. After sonication, the solutions were allowed to settle for 1 hour before measurement. A Cary 5000 UV-Vis NIR absorption spectrometer (Varian, USA) was used to confirm the dispersibility of the A-GNPs in each solution by evaluating the absorbance over a wavelength range of 220–800 nm.

To evaluate the solubility of A-GNPs, Hansen solubility parameters (HSPs), provide a useful framework to predict and improve the solubility, compatibility, stability and efficacy between the solvent and the solute [10]. The absorbance (A_{UV}) of a solution is closely related to the molar concentration (c), as expressed by the Beer-Lambert law,

$$A_{UV} = \varepsilon c l \tag{5}$$

where ε the molar absorptivity and l is the path length of a cell.

The Beer-Lambert law illustrates that a solution with a higher content of A-GNPs would absorb visible light and thus will become more opaque. An open-source Hansen solubility parameter estimation toolkit, MLoc (version 1.0), which is based on minimising the overall weighted distance between material (M) and solvents in the Hansen space, is used for HSP calculation. The HSP may be separated into three different types of partial solubility parameters related to the specific intermolecular interactions [11-13]:

$$\delta^2 = \delta_d^2 + \delta_p^2 + \delta_h^2 \tag{6}$$

where d, p, and h refer to the dispersion, polar, and hydrogenbonding interactions. The HSP Distance between two molecules, conventionally called Ra, is the measure of how alike they are. The smaller Ra, the more likely they are to be compatible. This well-known equation is expressed as,

$$Ra^2 = 4(\delta_{d1} - \delta_{d2})^2 + \left(\delta_{p1} - \delta_{p2}\right)^2 + (\delta_{h1} - \delta_{h2})^2 \tag{7}$$

Hansen solubility parameters for the organic solvents examined, and the distances between the solubility parameters of the A-GNPs and solvents (unit: $MPa^{1/2}$). The results are summarized in Table S4 and the 3D M Locator plot shown in Figure 4a.

Table S1: Selected solubility parameters calculated using Fedors' method.

Group	e_i	v_i
3 CH_3	3375	100.5
4 CH_2	4720	64.4
3 CH	2460	-3
2 NH_2	6000	38.4
2 O	1600	7.6
	18155	207.9

Table S2: Solubility parameter of each component.

Components		Chemical structure	Solubility parameter (MPa$^{1/2}$)
Epoxy resin	DGEBA	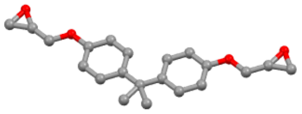	21.3
Hardeners	POPD	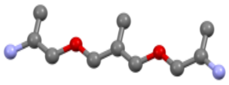	19.1
	IPDA	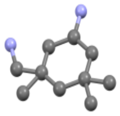	19.2
	AEPIP	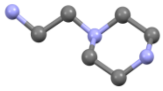	20.9

Table S3: Kissinger kinetic analysis for the amine-cured resin blends.

Blend	Kissinger method, E_a (kJ mol^{-1})	Pre-exponential factor, A_p (s^{-1})	Rate constant, k (10^{-2}) at 298 K (s^{-1})	Rate constant, k (10^{-2}) at 333 K (s^{-1})
DGEBA-POPD	55.9	6.1 x 10^7	1.0	10.3
DGEBA-IPDA	43.8	2.2 x 10^6	4.4	28.6
DGEBA-AEPIP	56.9	5.6 x 10^8	6.1	66.0

Table S4: Hansen solubility parameters for the organic solvents examined, and the distances between the solubility parameters of the A-GNPs and solvents (unit: $MPa^{1/2}$)

	δ_d	δ_p	δ_h	δ	Ra
A-GNPs	16.2	9.9	14.0	23.6	-
Isopropanol	15.8	6.1	16.4	23.5	4.5
DMF	17.4	13.7	11.3	24.9	5.3
Ethanol	15.8	8.8	19.4	26.5	5.6
Acetone	15.5	10.4	7	19.9	7.1
THF	16.8	5.7	8	19.5	7.4
Ethyl acetate	15.8	5.3	7.2	18.2	8.2
Methanol	15.1	12.3	22.3	29.6	8.9
Acetonitrile	15.3	18	6.1	24.4	11.5
Xylene	17.6	1	3.1	17.9	14.3
PC	20	18	4.1	27.2	14.9
Toluene	18	1.4	2	18.2	15.1
Hexane	14.9	0	0	14.9	17.3
Water	15.5	16	42.3	47.8	29.0

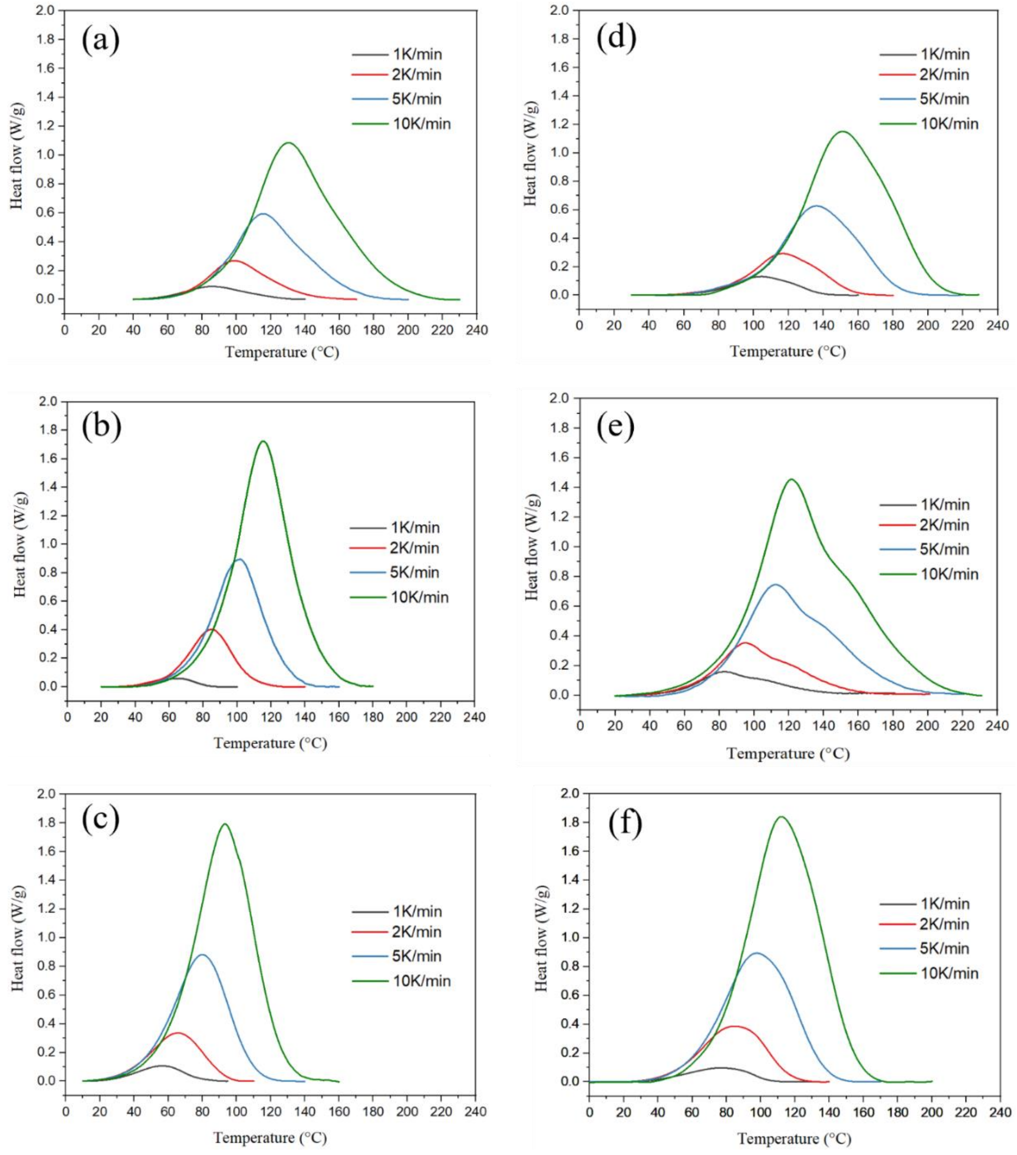

Figure S1: DSC thermograms (plotted as heat flow *versus* temperature with exotherms upwards) of resin blends 1 (a), 2 (b), and 3 (c) at several different heating rates: 1, 2, 5, 10°C min^{-1}.

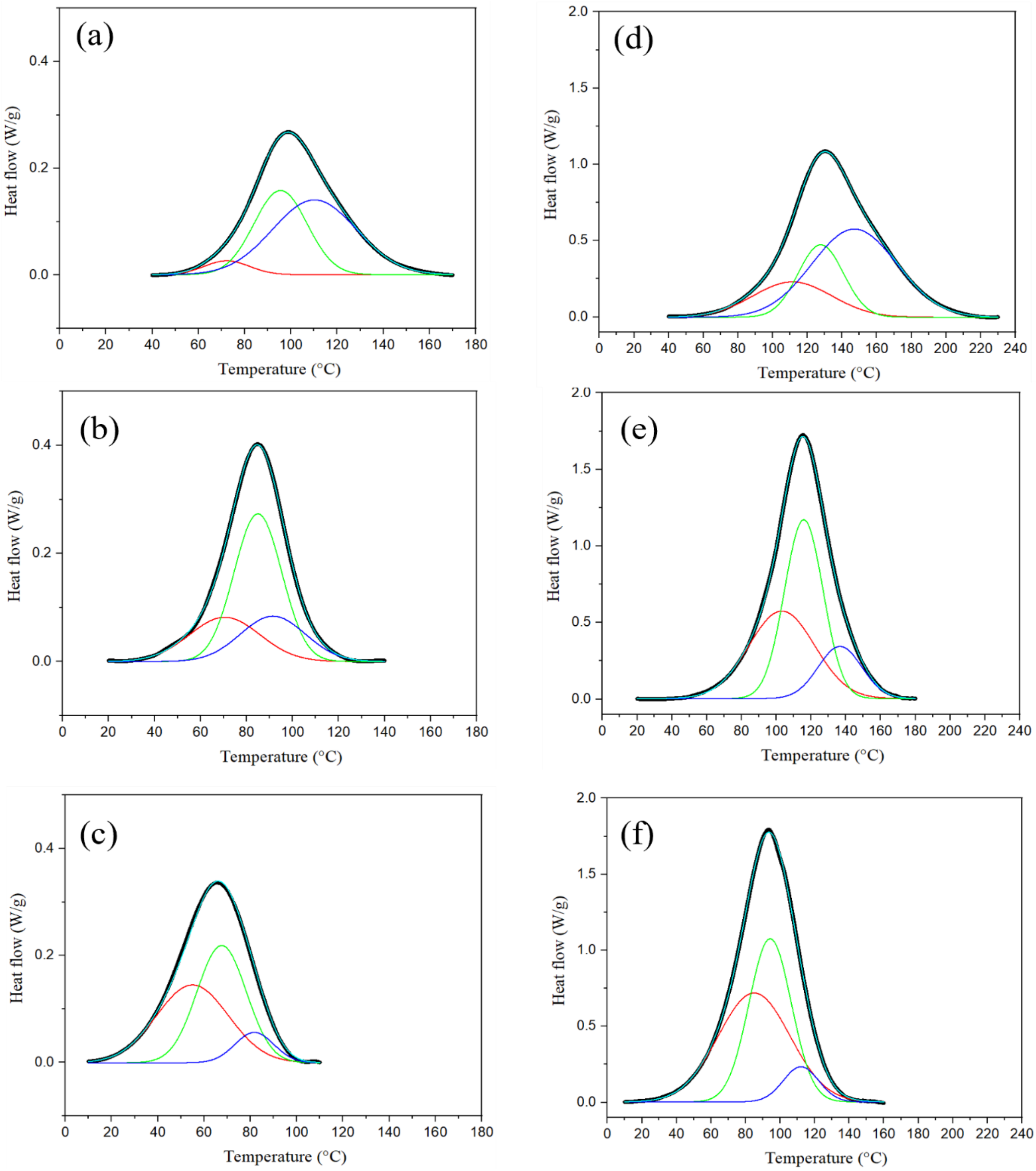

Figure S2: Peak fitting for DSC data (plotted as heat flow *versus* temperature with exotherms upwards) at 2 K/min and 10K/min (under nitrogen) for different blends: (a) DGEBA-POPD, (b) DGEBA-IPDA, (c) DGEBA-AEPIP.

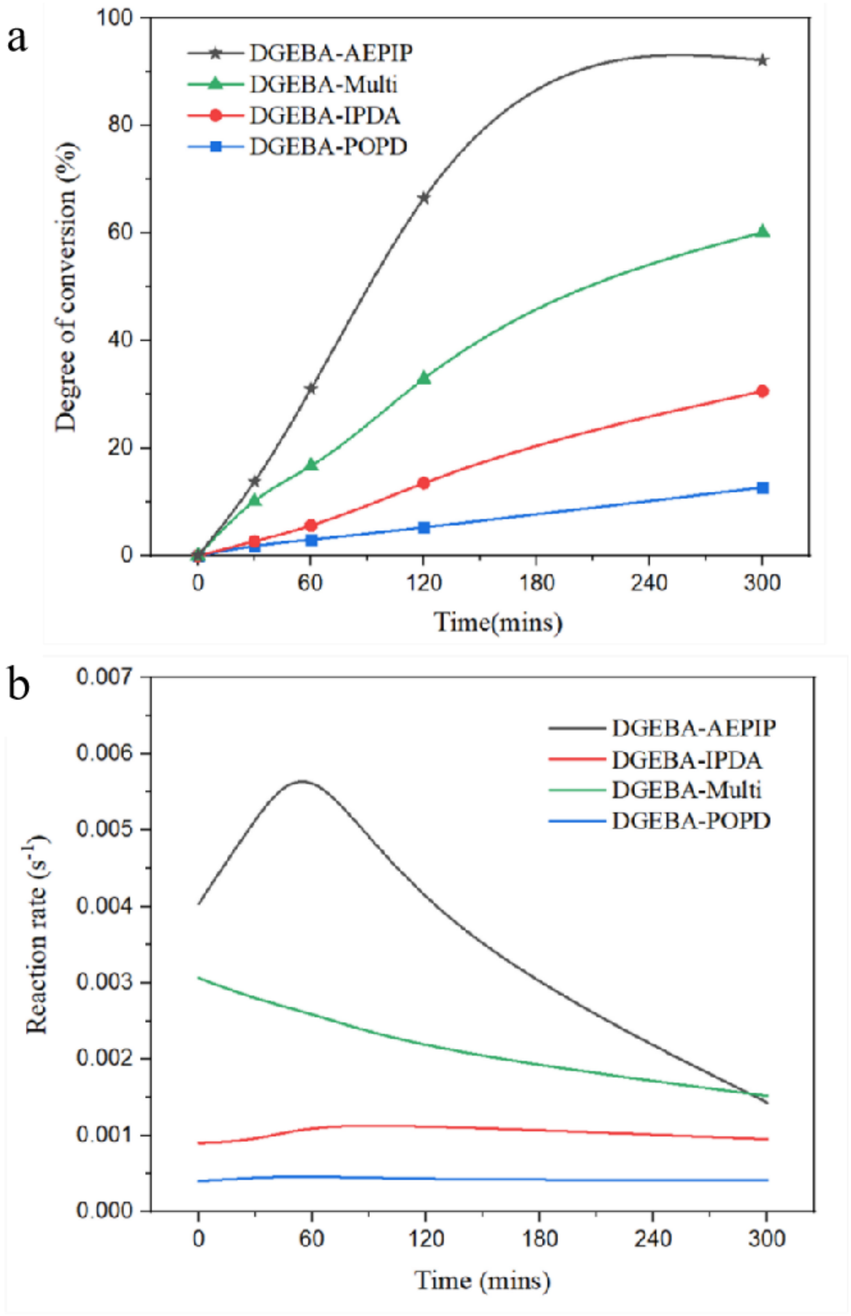

Figure S3: Plot of the degree of cure *versus* time (a) and reaction rate *versus* time for the isothermal cure at various temperatures.

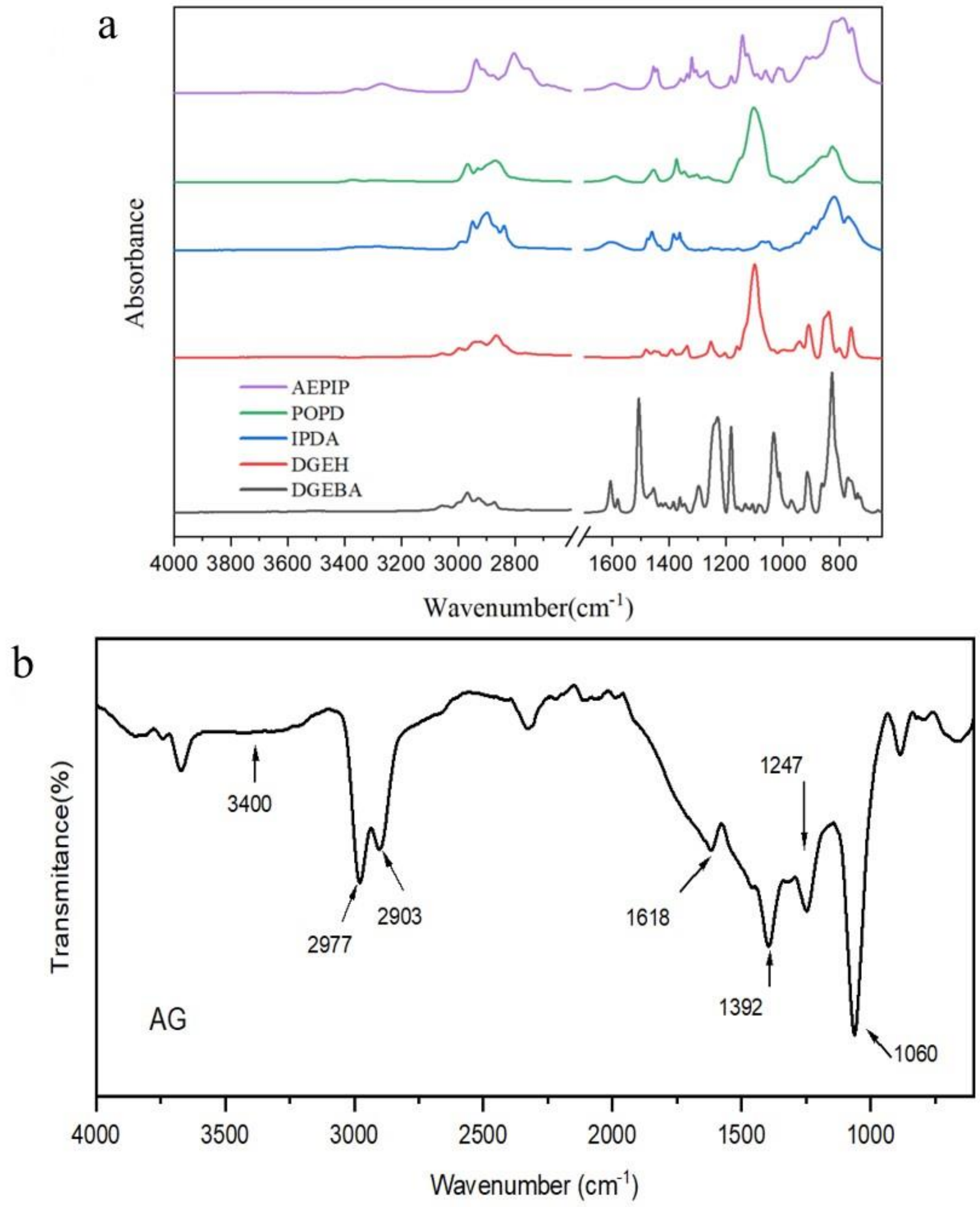

Figure. S4. Plots of FTIR spectra of a) chemical components shown plotted as absorbance *versus* wavenumber, and b) A-GNPs, plotted as transmittance *versus* wavenumber.

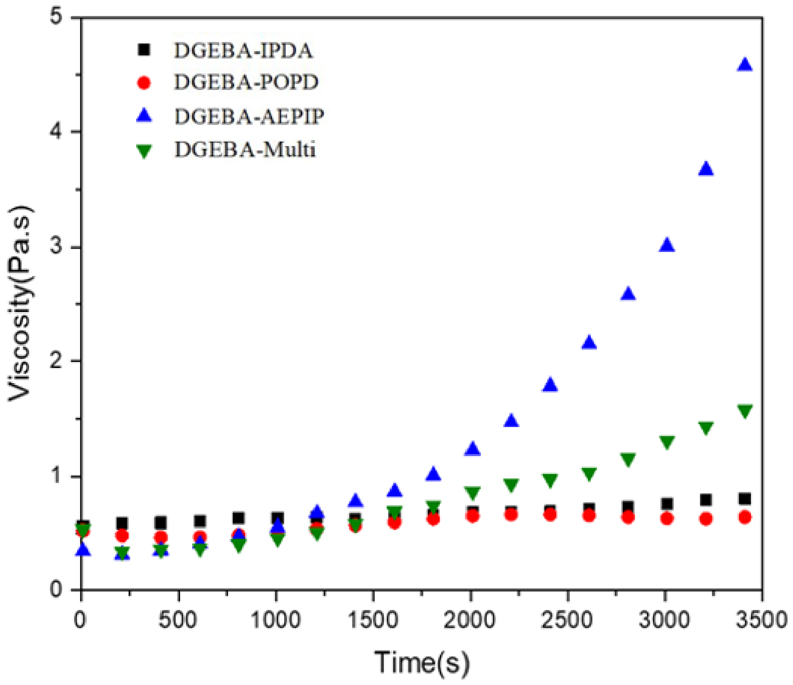

Figure S5. The plot of viscosity *versus* time for amine-cured resin blends during the early stages of polymerisation.

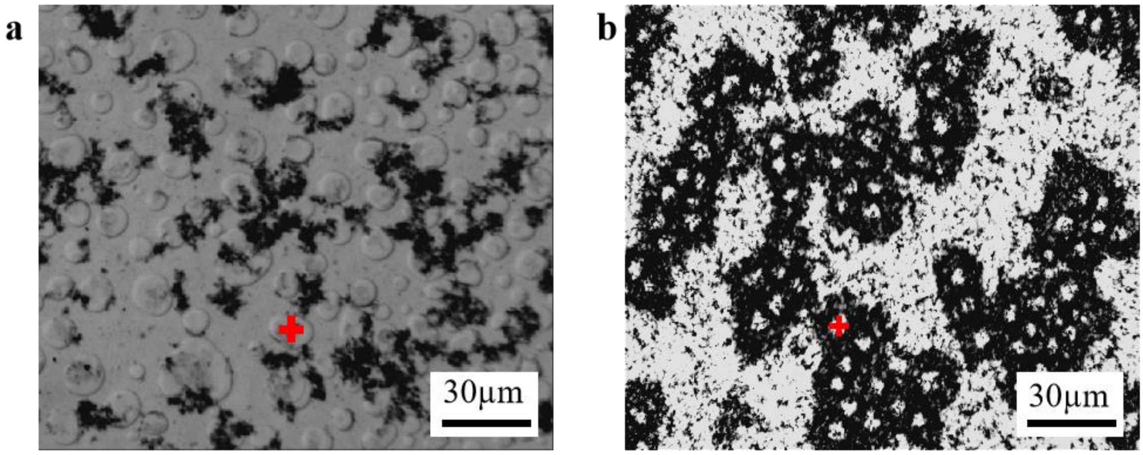

Figure S6. SEM data for a) epoxy/CNT, and b) epoxy/GNP nanocomposites blend of A-BC (3:7).

References

[1] A. Boudenne, L. Ibos, Y. Candau, S. Thomas, Handbook of Multiphase Polymer Systems, John Wiley & Sons, 2011.

[2] I. Hamerton, L.T. McNamara, B.J. Howlin, P.A. Smith, P. Cross, S. Ward, Examining the initiation of the polymerization mechanism and network development in aromatic polybenzoxazines, Macromolecules 46(13) (2013) 5117-5132.

[3] H.E. Kissinger, Variation of peak temperature with heating rate in differential thermal analysis, J Res Natl Bur Stand 57(4) (1956) 217-221.

[4] B.K. Russell, S. Takeda, C. Ward, I. Hamerton, Examining the influence of carboxylic anhydride structures on the reaction kinetics and processing characteristics of an epoxy resin for wind turbine applications, Reactive and Functional Polymers 144 (2019) 104353.

[5] B.K. Russell, Developing Capabilities in Materials and Manufacture for Wind Turbine Blades by the Application of an Anhydride-cured Epoxy Resin System, University of Bristol, 2021.

[6] R. Hardis, Cure Kinetics Characterization and Monitoring of an Epoxy Resin for Thick Composite Structures, Iowa State University, 2012.

[7] L. Khoun, T. Centea, P. Hubert, Characterization methodology of thermoset resins for the processing of composite materials—case study: CYCOM 890RTM epoxy resin, 44(11) (2010) 1397-1415.

[8] A. Tsiamis, R.J. Iredale, R. Backhouse, S.R. Hallett, I. Hamerton, Liquid processable, thermally stable, hydrophobic phenolic triazine resins for advanced composite applications, 1(6) (2019) 1458-1465.

[9] Chen J, Yan W, Townsend E J, *et al.* Tunable surface area, porosity, and function in conjugated microporous polymers. Angewandte Chemie International Edition, 2019, 58(34): 11715-11719.

[10] C.M. Hansen, Hansen Solubility Parameters: A User's Handbook, CRC Press, 2007.

[11] Xue Fang, Ulzhalgas Karatayeva, Ella M. Gale, Natalie Fey, Charl FJ Faul, MLoc (v1.0), Release, 2023-01, https://github.com/xueannafang/hsp_toolkit_prototype/tree/main/HSP_MLocator (accessed 2024-05-08).

[12] Xue Fang, Ulzhalgas Karatayeva, Ella M. Gale, Natalie Fey, Charl FJ Faul, MLoc – A semi-empirical solvent selection framework for porous organic materials (manuscript in preparation), 2024.

[13] Xue Fang, Ulzhalgas Karatayeva, Ella M. Gale, Natalie Fey, Charl FJ Faul, MLoc (v2.0), Release 2023-7, https://github.com/xueannafang/hsp_mloc_v2, (accessed 2024-05-08).
